# Supplementary material for: Evaluation of the B.strong Queensland Indigenous Health Worker Brief Intervention Training Program for Multiple Health Risk Behaviours
Source: Int J Environ Res Public Health. 2021 Apr 16;18(8):4220. doi: 10.3390/ijerph18084220 (PMC8073127; doi:10.3390/ijerph18084220)
Supplement: Supplementary file 1 [file ijerph-18-04220-s001.zip › ijerph-1155639-supplementary.pdf]

Supplementary Material, Figure S.1. B.strong Evaluation Logic Model

INPUTS

- Funding: Queensland Health Active Healthy Living
- Administering institution: Menzies School of Health Research
- Collaborators: Cancer Council Qld, The University of Queensland, Apunipima Cape York Health Council
- Key stakeholders: HHSs, QAIHC, ACCHSs, Education, Corrections, Indigenous primary healthcare services, Community partners, Quitline, Diabetes Qld

ACTIVITIES

- Project establishment and governance
- Literature review and environmental scan
- Evaluation and monitoring
- Program development
- Pilot testing
- Engagement of stakeholders
- State-wide implementation
- Health promotion and marketing

OUTPUTS

- Program Steering Group and Expert Advisory Group
- Indigenous Reference Group
- Terms of Reference/s
- Annual Forward Plans
- Engagement with HHSs and other services
- 3 year Evaluation and Monitoring plan
- Environmental scan of existing brief intervention programs undertaken
- Review of scientific literature on best practice completed
- Review of current guidelines across all key areas (tobacco, nutrition, physical activity) undertaken
- Review and adaption of existing training programs (i.e. *SmokeCheck*) undertaken
- Review and adaption of client resources completed
- Review and adaption of practitioner materials
- Development of eModules (tobacco, nutrition, physical activity)
- Pilot testing of F2F training program
- Evaluation of F2F pilot testing
- Pilot testing of eModules
- Evaluation of eModules pilot testing
- Quarterly and annual reporting
- Management briefings and Community engagement
- Signed commitment for practitioners to attend training
- State-wide rollout of F2F training workshops
- State-wide rollout of eModules
- Peer support program and Help Desk established to support implementation
- Community of Practice (Facebook) established
- Certificates provided to participants for CPD points
- Promotional materials disseminated
- Co-branding opportunities identified

OUTCOMES

- Delivery of B.strong workshops to 15 geographical HHSs
- 1131 health practitioners trained to deliver brief interventions for tobacco cessation, nutrition and physical activity, including 360 completing online modules
- Increased documentation of brief interventions undertaken in health services and recorded in the service's patient records
- Provision of post-training support to practitioners
- Evidence of increased knowledge, skills, confidence and competence in delivery of brief interventions by health practitioners
- Increased referral rates to specialist programs such as Quitline
- Evidence of increased awareness of healthy lifestyle behaviours and detriments of tobacco smoking, poor nutrition, inactivity and chronic illness

AIM:

To develop, promote, implement and evaluate the B.strong Program

OBJECTIVES:

- Increase access by Indigenous health workers to appropriate brief intervention training
- Increase delivery of brief interventions to Indigenous clients in primary and community settings
- Increase referral of Indigenous clients to screening and early intervention programs and services
- Increase knowledge and awareness of key risk factors for chronic disease in Indigenous communities

Reduced morbidity and mortality

- Improvement in rates of non-smokers
- Improvement in rates of Indigenous people in the healthy weight range
- Increased awareness of benefits of physical activity
- Decreased rates of chronic illness (e.g. diabetes, heart disease etc.)

Table S.1. Post-workshop/3-month follow-up comparisons

Domain	N of pairs	Post WS mean (SD)	3-month FU WS mean (SD)	Mean diff. (95% CI)	t	*p value*
**Knowledge: h**ow do you rate your knowledge of the impact on chronic illness of:						
smoking	142	4.11 (0.754)	4.08 (0.773)	-0.035 (-0.189 – 0.118)	-0.453	0.651
nutrition	141	4.11 (0.753)	4.05 (0.720)	-0.057 (-0.203 – 0.089)	-0.769	0.443
physical activity	141	4.13 (0.764)	4.09 (0.702)	-0.043 (-0.189 – 0.104)	-0.576	0.566
**Confidence (1):** How confident are you in talking with your clients about their:						
smoking	210	4.10 (0.807)	4.00 (0.899)	-0.100 (-0.214 – 0.014)	-1.729	0.085
nutrition	208	4.13 (0.819)	4.05 (0.838)	-0.077 (-0.187 – 0.033)	-1.375	0.171
physical activity	208	4.14 (0.813)	4.04 (0.853)	-0.096 (-0.214 – 0.022)	-1.607	0.109
**Confidence (2):** How confident are you in assessing your clients' readiness to:						
quit smoking	210	4.01 (0.815)	3.90 (0.901)	-0.119 (-0.247 – 0.009)	-1.839	0.067
improve nutrition	210	4.07 (0.816)	3.90 (0.841)	-0.171 (-0.288 – -0.055)	-2.896	0.004
increase physical activity	210	4.08 (0.815)	3.90 (0.847)	-0.171 (-0.294 – -0.049)	-2.756	0.006
**Attitudes**						
Participants have a clear idea of their responsibilities in helping clients with health behaviour change.	141	4.46 (0.567)	4.38 (0.604)	-0.085 (-0.191 – 0.021)	-1.584	0.115
Participants feel there is little they can do to help clients change their health behaviours.	142	2.30 (1.293)	2.31 (1.143)	0.014 (-0.226 – 0.254)	0.116	0.908
Participants feel uncomfortable asking clients about their health behaviours.	142	2.44 (1.252)	2.54 (1.241)	0.099 (-0.156 – 0.353)	0.765	0.446
**Practices (1):** how often do you ask clients about their:						
smoking habits	97	4.40 (0.731)	3.93 (0.992)	-0.474 (-0.644 – -0.304)	-5.542	0.000
nutrition habits	97	4.42 (0.626)	4.01 (0.797)	-0.412 (-0.571 – -0.254)	-5.159	0.000
physical activity.	97	4.40 (0.717)	3.92 (0.874)	-0.485 (-0.641 – -0.328)	-6.128	0.000

**Usual practices (2):** How often do you provide brief interventions to clients relating to:						
smoking	96	2.46 (0.521)	2.30 (0.583)	-0.156 (-0.279 – -0.034)	-2.535	0.013
nutrition	96	2.49 (0.503)	2.33 (0.496)	-0.156 (-0.279 – -0.034)	-2.535	0.013
physical activity.	96	2.49 (0.523)	2.33 (0.536)	-0.156 (-0.279 – -0.034)	-2.535	0.013

Table S.2. Pre-workshop/3-month follow-up comparisons

Domain	N of pairs	Pre WS mean (SD)	3 mth FU mean (SD)	Mean diff. (95% CI)	t	*p value*
**Knowledge: h**ow do you rate your knowledge of the impact on chronic illness of:						
smoking	145	3.48 (0.906)	4.05 (0.785)	0.566 (0.396 - 0.735)	6.593	0.000
nutrition	145	3.54 (0.834)	4.02 (0.731)	0.483 (0.330 - 0.635)	6.259	0.000
physical activity	145	3.59 (0.854)	4.06 (0.715)	.462 (0.304 - 0.620)	5.767	0.000
**Confidence (1):** How confident are you in talking with your clients about their:						
smoking	213	336 (1.106)	398 (0.901)	0.620 (0.480 - 0.759)	8.756	0.000
nutrition	213	3.46 (1.113)	4.03 (0.841)	0.573 (0.433 - 0.713)	8.059	0.000
physical activity	213	3.48 (1.093)	4.03 (0.852)	0.549 (0.410 - 0.689)	7.752	0.000
**Confidence (2):** How confident are you in assessing your clients' readiness to:						
quit smoking	213	3.00 (1.122)	3.89 (0.894)	0.892 (0.737 - 0.1047)	11.358	0.000
improve nutrition	213	3.14 (1.050)	3.89 (0.834)	0.746 (0.606 - 0.887)	10.452	0.000
increase physical activity	213	3.19 (1.084)	3.90 (0.840)	0.704 (0.560 - 0.848)	9.652	0.000
**Attitudes**						
Participants have a clear idea of their responsibilities in helping clients with health behaviour change.	144	4.00 (0.729)	4.37 (0.600)	0.368 (0.232 - 0.504)	5.349	0.000
Participants feel there is little they can do to help clients change their health behaviours.	144	2.51 (0.961)	2.31 (1.137)	1.201 (-0.409 - 0.006)	-1.917	0.057
Participants feel uncomfortable asking clients about their health behaviours.	144	2.63 (1.002)	2.54 (1.234)	-0.090 (-0.303 - 0.122)	-0.840	0.402
**Practices (1):** how often do you ask clients about their:						
smoking habits	139	3.67 (1.125)	4.04 (0.947)	0.374 (0.212 - 0.536)	4.570	0.000
nutrition habits	139	3.85 (0.970)	4.05 (0.862)	0.201 (0.047 - 0.355)	2.586	0.011
physical activity.	139	3.83 (0.947)	4.02 (0.872)	0.194 (0.043 - 0.345)	2.545	0.012

**Usual practices (2):** How often do you provide brief interventions to clients relating to:						
smoking	86	201 (0.584)	2.31 (0.599)	0.302 (0.166 - 0.438)	4.424	0.000
nutrition	86	2.05 (0.529)	2.34 (0.500)	0.291 (0.164 - 0.418)	4.558	0.000
physical activity.	86	2.12 (0.495)	2.36 (0.507)	0.244 (0.117 - 0.371)	3.820	0.000

Notes:

Knowledge and confidence: improvement statistically significant in all domains.
Attitudes: improvement not sustained in two out of the three domains.
Practices: improvements sustained, statistically significant in all three domains.
